# Ordering a rhenium catalyst on Ag(001) through molecule-surface step interaction

**DOI:** 10.1038/s42004-021-00617-9

**Published:** 2022-01-10

**Authors:** Ole Bunjes, Lucas A. Paul, Xinyue Dai, Hongyan Jiang, Tobias Claus, Alexandra Rittmeier, Dirk Schwarzer, Feng Ding, Inke Siewert, Martin Wenderoth

**Affiliations:** 1 IV. Physikalisches Institut, Georg-August-Universität Göttingen, Friedrich-Hund-Platz 1, 37077 Göttingen, Germany; 2 Institut für Anorganische Chemie, Georg-August-Universität Göttingen, Tammannstraße 4, 37077 Göttingen, Germany; 3 Center for Multidimensional Carbon Materials, Institute for Basic Science (IBS), Ulsan, 44919 Republic of Korea; 4 Department of Dynamics at Surfaces, Max-Planck Institute for Biophysical Chemistry, Am Faßberg 11, 37077 Göttingen, Germany; 5 Department of Materials Science and Engineering, Ulsan National Institute of Science and Technology (UNIST), Ulsan, 44919 Republic of Korea

**Keywords:** Molecular self-assembly, Scanning probe microscopy, Surface assembly, Density functional theory

## Abstract

Atomic scale studies of the anchoring of catalytically active complexes to surfaces may provide valuable insights for the design of new catalytically active hybrid systems. In this work, the self-assembly of 1D, 2D and 3D structures of the complex *fac*-Re(bpy)(CO)_3_Cl (bpy = 2,2′-bipyridine), a CO_2_ reduction catalyst, on the Ag(001) surface are studied by a combination of low-temperature scanning tunneling microscopy and density functional theory calculations. Infrared and sum frequency generation spectroscopy confirm that the complex remains chemically intact under sublimation. Deposition of the complexes onto the silver surface at 300 K leads to strong local variations in the resulting surface coverage on the nanometer scale, indicating that in the initial phase of deposition a large fraction of the molecules is desorbing from the surface. Low coverage regions show a decoration of step edges aligned along the crystal’s symmetry axes <110>. These crystallographic directions are found to be of major importance to the binding of the complexes to the surface. Moreover, the interaction between the molecules and the substrate promotes the restructuring of surface steps along these directions. Well-aligned and decorated steps are found to act as nucleation point for monolayer growth (2D) before 3D growth starts.

## Abstract (web summary)

Catalytically active complexes adsorbed to noble metal surfaces hold potential for CO_2_ utilisation. Here, the mechanism of growth of *fac*-Re(bpy)(CO)_3_Cl on Ag(001) is shown to begin at well-oriented surface steps, with surface restructuring promoting the growth of long-range ordered assemblies.

## Introduction

In order to reach CO_2_ neutrality, catalytically active molecules that convert CO_2_ to valuable products have been in the focus of research for decades^1^. Besides optimizing the catalytic activity^2^, one of the goals is to develop and to design new catalytically active hybrid systems, e.g. molecules adsorbed on a surface to combine the advantages of molecular systems, e.g. in selectivity, with the advantages of heterogeneous systems^3–6^. Anchoring the molecules to a surface can result in a well-defined geometry/orientation of the molecules to a liquid or a gas phase eventually providing a defined catalytically active interface. A large-scale ordered structure can be realized by molecular self-assembly^7^, which also provides access to a well-defined playground for tailoring the molecular reactivity within a specific local environment^8^.

Self-organization of molecular clusters, also fundamental in the fields of molecular electronics and sensing technology, has attracted a lot of attention^9–11^. In this context, the role of surface defects can be very contradictory. For example, on the one hand, surface steps acting as grain boundaries^12^ can prevent long-range order across different substrate terraces. On the other hand, steps have shown potential to be used as selective adsorption sites^13^ as well as to induce rotational alignment of molecules^14,15^. And, applying multi-step preparation techniques, e.g. thermally induced polymerization causing an alignment of the step edges, the prepared step edges can be used to steer molecular layer growth^16,17^. To find suitable combinations of surfaces and molecules, one has (a) to establish appropriate preparation methods, (b) to prove chemical stability of the molecule on the surface as well as (c) to understand the interaction of the molecules with the surface and its defects in order to finally control the growth of 1D (molecular wires), 2D (monolayers) and 3D molecular structures^15,18^.

**Fig. 1:**
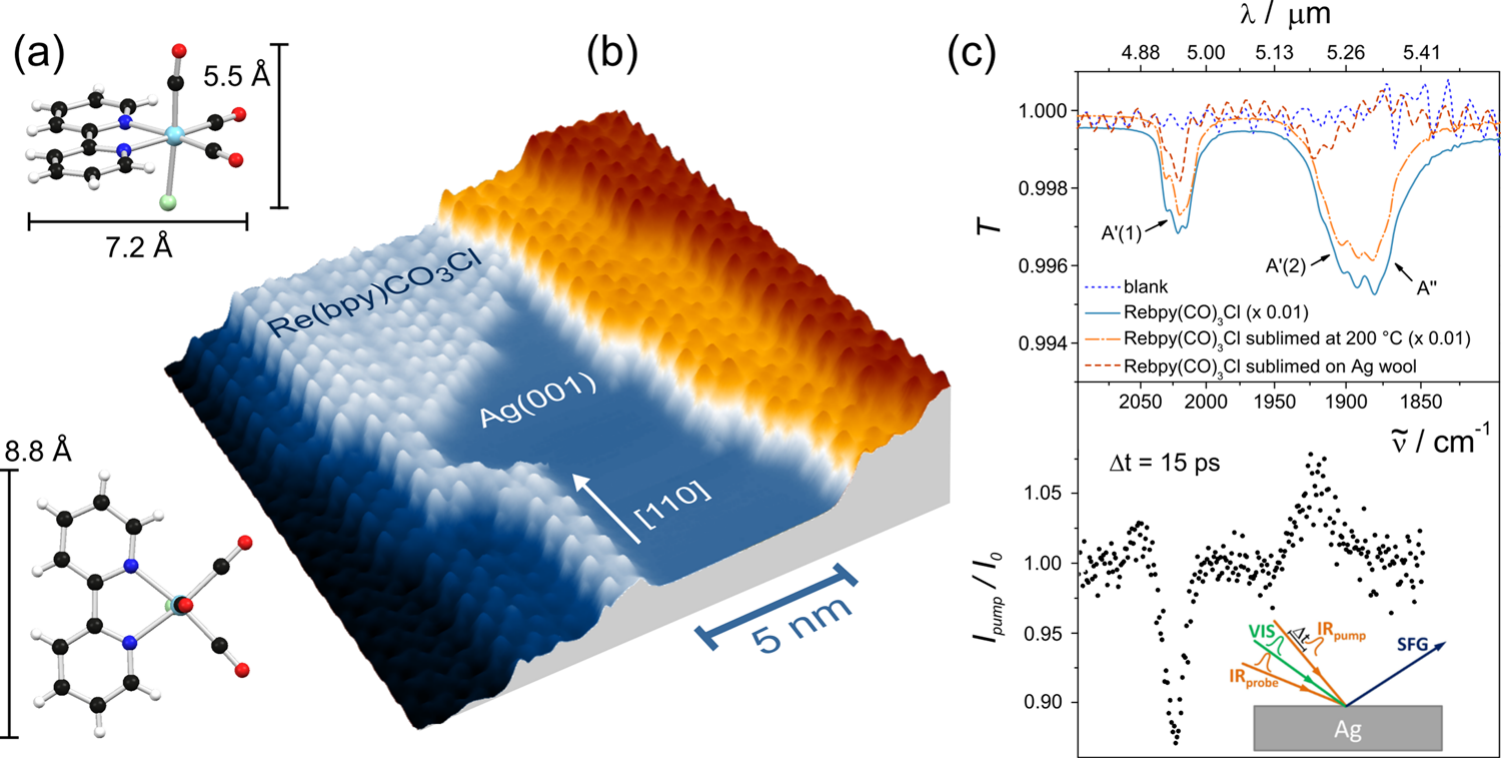
Growth of *fac*-Re(bpy)(CO)_3_Cl (bpy = 2,2′-bipyridine) on Ag(001). **a** The structure of the complex as a ball-and-stick model in top and side view^44,45^. The colors of the balls represent atoms of different elements: black = C, white = H, red = O, blue = N, green = Cl, turquoise = Re. **b** High resolution topography showing self-assembled molecular structures on a stepped Ag(001) surface, (*I*_set_ = 50 pA, *U*_bias_ = 0.4 V and *T* = 80 K). The coverage of the individual terraces is inhomogeneous. Molecular chains are found at monoatomic step edges oriented along the crystal direction [110] of the silver substrate (indicated by the white arrow). **c** Top: Ex-situ IR spectra of *fac*-Re(bpy)(CO)_3_Cl powder as KBr pellets directly after the synthesis (light blue), after sublimation of the complex at *T* = 200 °C and with *p* < 10^–3^ mbar (orange), and after sublimation onto silver wool (red). The shown wavenumber regime with the indicated CO vibrational modes is used to monitor the chemical stability of the complex^46^. Bottom: IR_pump_ pulse induced change in SFG response of the molecular monolayer covered crystal surface at 300 K. One can clearly identify CO stretching bands at 1920 and 2025 cm^−1^. The inset shows the measurement scheme.

In this study, we have investigated the growth of *fac*-Re(bpy)(CO)_3_Cl (bpy = 2,2′-bipyridine), notably a three-dimensional complex, on the noble metal surface Ag(001) using scanning tunneling microscopy (STM). The complex *fac*-Re(bpy)(CO)_3_Cl (bpy = 2,2′-bipyridine), a 3D depiction is shown in Fig. 1a, is a well-known photo- and electrochemical CO_2_-reduction catalyst^19^. Several groups have already shown interest in tuning the molecule’s structure^20^ and in mimicking its properties in larger complexes to be anchored on certain surfaces^4,21–23^. In addition, studies of the electro-reduction products of a related complex on graphite^24^ and the covalent binding of a different related complex to TiO_2_^25^ were reported.

We report strong evidence that the growth at room temperature predominantly starts at well-oriented steps. Furthermore, molecular-induced surface restructuring promotes the growth of self-assembled molecular structures. Density functional theory (DFT) provides the basis for a comprehensive understanding of growth.

## Results

### Stability of the complex during the sample preparation procedure

Molecules containing CO ligands are known to easily lose CO ligands during heating procedures or upon irradiation^26^. Moreover, ligand dissociation upon adsorption of the three-dimensional complex on the flat surface cannot be excluded a priori. Thus, we tested the thermal stability of the complex by sublimation at temperatures of 200 °C using ex-situ infrared (IR) spectroscopy. In Fig. 1c the IR spectra are shown directly after synthesis (light blue), and after sublimation (orange). The characteristic CO vibrational bands stay invariant under sublimation indicating that the complex is unaffected by the heating procedure and stays chemically intact. In a second step, we tested the stability of the complex once it has been deposited on silver wool (details of the preparation can be found in Supplementary Note 1) serving as a multi-faceted model surface.

The IR spectrum of the washed-out and dried material is shown in Fig. 1c in red. Due to the small amount of complexes sublimed, the IR absorbance is rather low. For comparison, a spectrum of a blank sample is given in blue. The IR data show the characteristic high-energy CO-vibrations at 2020 cm^−1^. If the chloride ion dissociates, we would expect the formation of either [Re(bpy)(CO)_3_(solvent)]^+^ or [Re(bpy)(CO)_3_]_2_ after dissolution, which exhibit characteristic IR bands at ~2050 and ~1950 cm^−1^, respectively^27^. Neither of these bands is present in the IR spectra, indicating that the molecules stay intact upon deposition on randomly oriented silver.

The lower panel of Fig. 1c shows the normalized IR_pump_ induced sum-frequency generation (SFG) spectrum of a monolayer *fac*-Re(bpy)(CO)_3_Cl on a Ag(100) surface. The spectrum shows two features at 1920 ± 5 and 2025 ± 3 cm^−1^ which agree well with the IR spectrum of the carbonyl stretching bands of the solid material (upper panel in Fig. 1c). The conventional SFG spectrum of the A’(1) band, as described in Supplementary Note 2, gives a center frequency of 2033 ± 6 cm^−1^ consistent with Fig. 1c, too. These data support that the complex stays intact within the self-assembled molecular monolayers on the oriented silver surface.

Figure 1b shows a high-resolution topography mapping several silver terraces covered by a sub-monolayer of *fac*-Re(bpy)(CO)_3_Cl. While the complexes are found to form well-ordered structures, the coverage of the individual terraces is surprisingly inhomogeneous. In contrast to all the other terraces visible, the center terrace is only partially covered with complexes, although the incoming molecular flux is homogeneous on the given length scales. Topographies acquired on surfaces with small molecular coverage (Fig. 1b) show that even large molecular areas can coexist with Ag terraces, of which only the steps are decorated by molecular structures. However, only very rarely do we observe single isolated molecules and never molecular clusters on the free Ag surface.

While IR and SFG already strongly support the fact that the surface is covered by chemically intact molecules, STM can be used to search for local variations. Careful investigating of the molecular layers with small bias voltages and small tunnel currents revealed that none of the acquired data show any indication of different species being present on the surface. In other words, we do not have evidence for any chemical modification, e.g. ligand dissociation, of the complex on the surface after deposition.

### Growth of molecular clusters on Ag(001)

One central finding of this study is that the occupied step edges show a clear preferential orientation, namely the crystal direction [110] of the silver substrate, as can be seen in Fig. 1b. This stands in direct contrast to the pristine silver surface that does not show any preferential alignment of the steps (Supplementary Fig. 2).

**Fig. 2:**
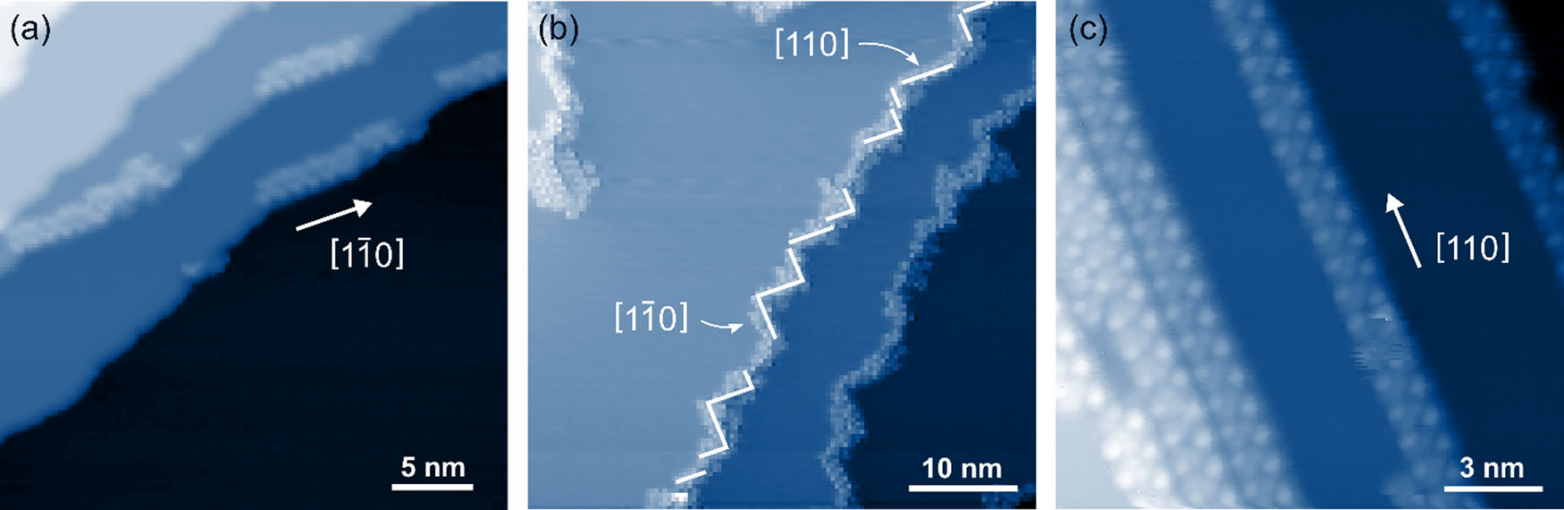
Preferential orientation found for step edges covered by molecules. **a** Topography of several steps edges that are barely covered by molecules. The occupied segments are aligned along the [110] direction of the substrate. **b** Topography shows several silver steps that are fully decorated by molecules. The step edges exhibit a predominant orientation along the [110] or the geometrically equivalent $[1\bar 10]$ crystal direction of the silver substrate. The step edge regions being aligned along these directions are indicated by white lines for one step edge. **c** Topography shows steps perfectly aligned along the substrate direction [110] that are fully decorated by molecules and show a periodic pattern. Tunneling parameters of all topographies were *I*_set_ = 50 pA, *U*_bias_ = 2 V and *T* = 80 K.

Figure 2a shows an example, in which small clusters at step edges are separated by randomly oriented unoccupied step edge regions. Crystallographic analysis of the occupied sections reveals a preferred step orientation, the direction $[1\bar 10]$, equivalent to [110]. Also, in the case of fully decorated step edges, the local preferential orientation can be seen as is illustrated by white lines in Fig. 2b. Many of the aligned segments have a length of a few nanometers.

Figure 2c shows that these ordered segments may extend to several tens of nanometers in this way forming long-range ordered 1D molecular wires. For a surface that, after deposition, was stored at room temperature for several days we even find ordered 1D wires as long as 100 nm (cf. Supplementary Fig. 3).

**Fig. 3:**
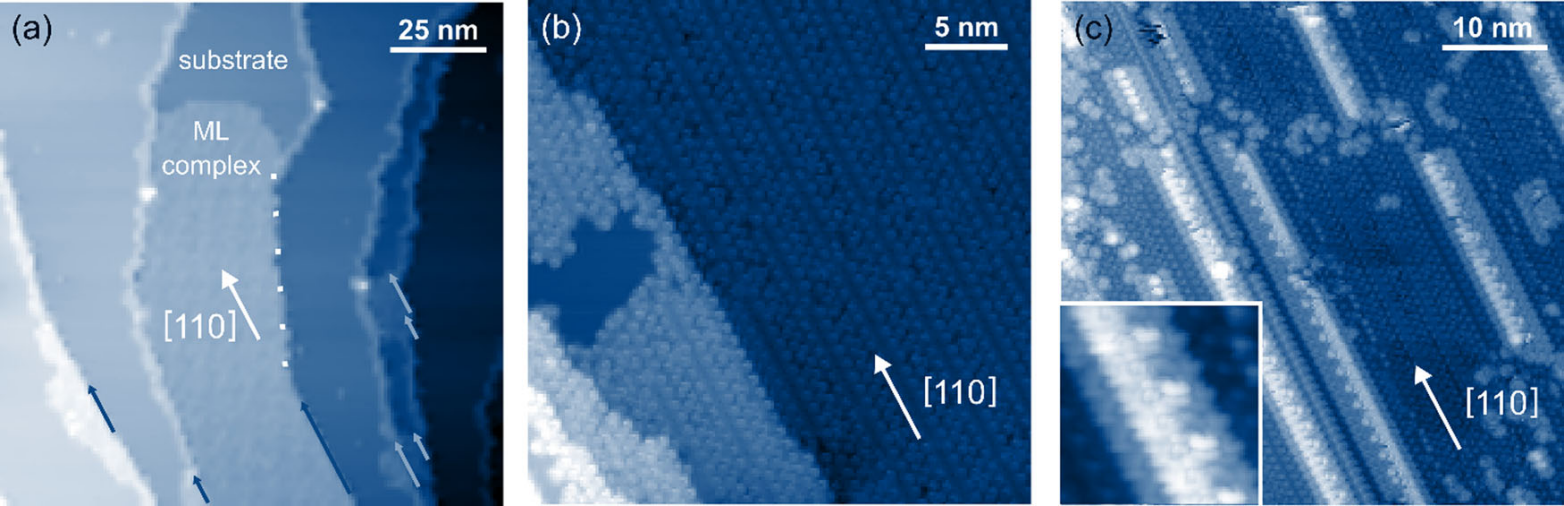
Growth of 2D and 3D molecular clusters influenced by substrate crystal direction [110]. **a** Topography (*T* = 80 K) showing several step edges being fully covered by molecules. Molecular monolayer growth is found for step edges being aligned along with the directions [110], indicated by light and dark blue arrows, and along [210] (dotted white line), cf. Supplementary Fig. 10. **b** Topography (*T* = 8 K) of a region with perfectly aligned step edges that show almost complete monolayer coverage. **c** Topography (*T* = 8 K) of 3D cluster growth. The molecular islands growing on top of the monolayer adopt the geometric orientation along [110]. The inset shows a high-resolution topography (6 × 6 nm²) of a molecular island showing a gear-rail-like structure on top of the island. The tunneling parameters for all topographies were *I*_set_ = 50 pA and *U*_bias_ = 2 V.

In the large-scale topography in Fig. 3a the transition from 1D growth at the steps to 2D growth on the terraces is shown. All the step edges are decorated by molecules. While on the center terrace full monolayer coverage is found, two-thirds of the image shows unoccupied silver. Small 2D monolayer areas are found at the positions indicated by the light and dark blue arrows. All 2D areas adjoin step edges that are well-aligned along the crystal direction [110] for several nanometers. In addition, a monolayer grows exclusively on the upper part of the steps, i.e. no growth is observed on the bottom of a step. This is visible, e.g., for the monolayer in the lower left-hand corner of Fig. 3a, dark blue arrow, that solemnly attaches to the lower flanking step of the silver terrace.

Figure 3b shows a large-scale topography of a nearly perfectly ordered monolayer. Such a complete monolayer occupation of the surface is mainly found in regions, in which the step edges are close to perfectly aligned along the silver crystal’s high symmetry axes 〈110〉.

For higher surface coverage, molecular islands are found on top of the monolayer. This can be seen in Fig. 3c. Moreover, for the growth of three-dimensional structures, the same 〈110〉 crystal directions have been found to have a major impact on structure growth. The islands clearly adopt the underlying monolayer geometry and, thus, are aligned along with the directions 〈110〉. In the high-resolution images in the inset of Fig. 3c the islands appear to have a base plateau of about 3 nm width. On top of this, a gear rail-like structure can be seen.

### DFT calculations of a single complex on Ag(001)

**Fig. 4:**
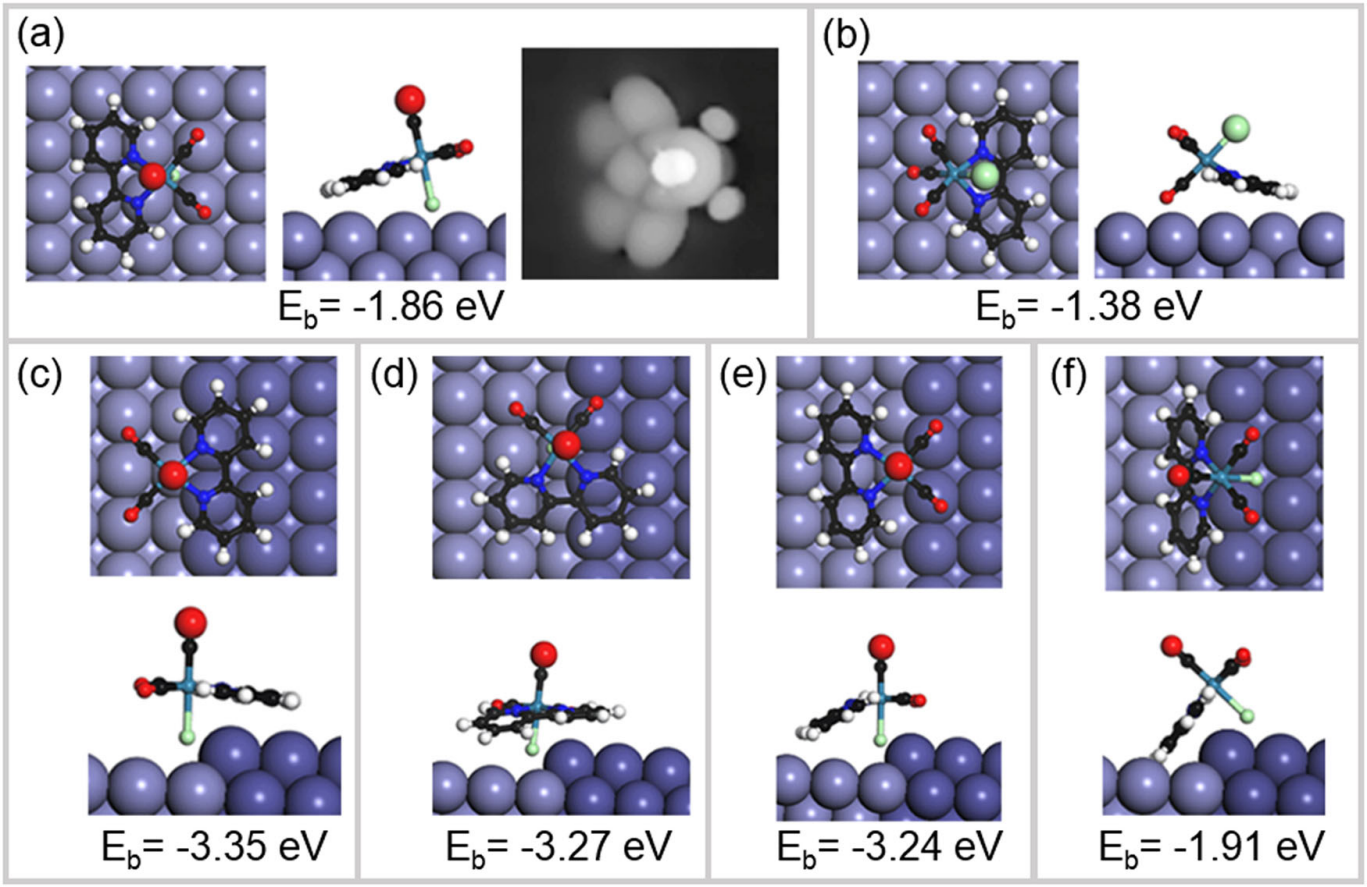
DFT simulations of a single rhenium complex on the Ag(001) surface. **a** Ball-and-stick model in top view and side view with chloride ion facing the surface as well as the simulated constant current topography, *U*_bias_ = 2 V. **b** Ball-and-stick model in top view and side view of a molecule with CO group facing the surface. **c**–**f** Four different geometric configurations of a single complex attached to a monoatomic silver step edge that is aligned along the crystal direction [110] in top view and side view. Below the belonging binding energies, *E*_b_, are shown.

**Fig. 5:**
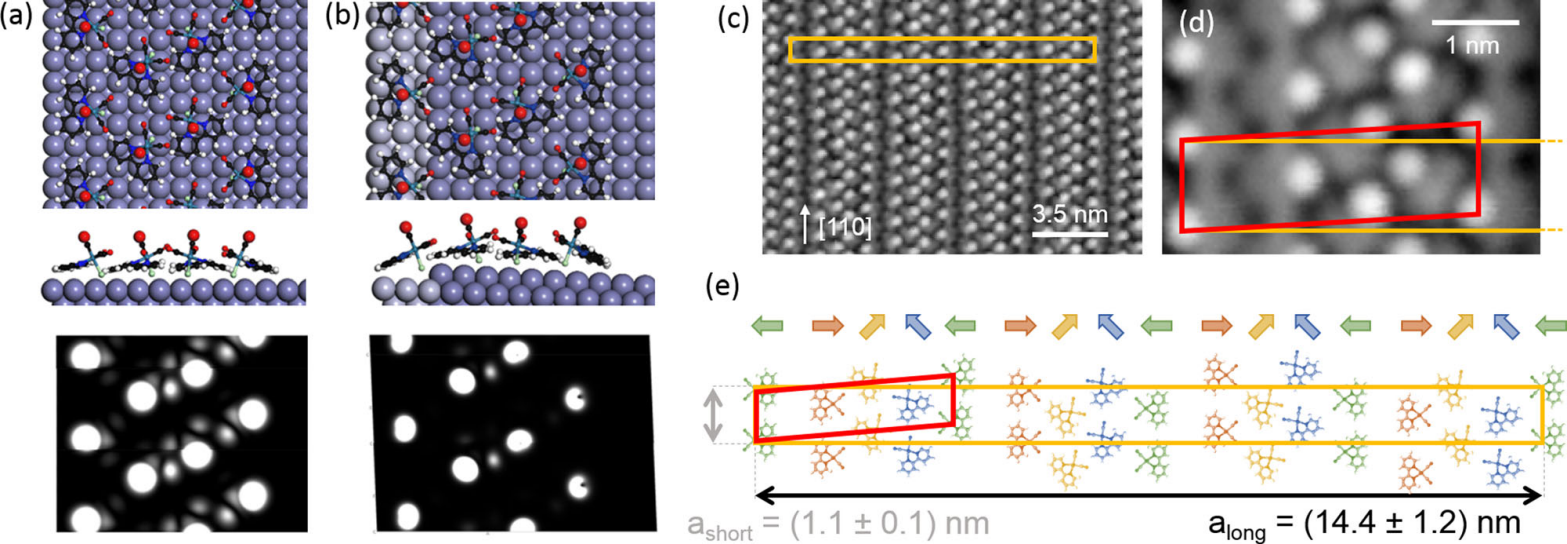
Model of the structure underlying the molecular monolayers. **a**, **b** Simulation of the molecular monolayer on the terrace and step edge in top view, side view, and the simulated STM images, *U*_bias_ = 2 V. **c** High-resolution topography of the molecular monolayer on the flat silver surface. While the primitive unit cell is depicted in red, the conventional unit cell that resembles the underlying geometry by taking into account the quadratic structure of the silver lattice is shown in orange. **d** High-resolution topography giving access to the orientation of the molecules within the primitive unit cell, framed in red. For comparison, the conventional unit cell is indicated in orange. **e** A simple model showing the orientation of the molecules within the conventional unit cell as identified on basis of STM data. The primitive and the conventional unit cell are framed in red and orange, respectively. The tunneling parameters for all topographies were *T* = 80 K, *I*_set_ = 50 pA, and *U*_bias_ = 1 V.

Starting the DFT analysis with a single *fac*-Re(bpy)(CO)_3_Cl molecule on a flat Ag(001) surface, we have considered two fundamentally different configurations: One in which the Cl atom faces the Ag(001) surface and the other one with the opposing CO group towards the Ag(001) surface. The DFT results show that the configuration with the Cl atom towards the Ag(001) surface has a binding energy of −1.86 eV, which is significantly lower than that with the CO group towards the Ag(001) surface, −1.38 eV (Fig. 4a, b). Comparing the experimental findings, e.g. Fig. 5d, with the simulated constant current STM topography of a single *fac*-Re(bpy)(CO)_3_Cl molecule in Fig. 4a supports this result. Consequently, analyzing more complex molecular structures, we have focused on configurations with the Cl atom facing the Ag(001) surface.

In the next step, triggered by the experimental findings, we have investigated a single *fac*-Re(bpy)(CO)_3_Cl molecule attached to a monatomic [110] step edge. Figure 4c–f shows different configurations with binding energy being greatly decreased up to *E*_b_ = −3.35 eV with respect to the free surface. Careful exploration of the three energetically most favorable *fac*-Re(bpy)(CO)_3_Cl configurations on the step edge shows that the respective binding energy differs only by about ~0.1 eV (Fig. 4c–e). In Fig. 4f the local environment of the Cl ligand is comparable to the local environment of the molecule on the undisturbed surface, shown in Fig. 4a. Both configurations are attributed very similarly but higher binding energies compared to the configurations Fig. 4c–e. Hence, we attribute the strong decrease in the binding energy to the interaction between the Cl ligand and the step edge. In general, the binding of the chloride ion to the silver surface is accompanied by significant charge transfer. At the step edge, additional charge transfer occurs between the molecule and the nearby step atoms leading to a stronger chemical bond compared to the molecule on the free surface. The charge transfer becomes evident, e.g., by the Bader charges calculated for the chloride ion changing from −0.62e (the molecule in vacuum) over −0.56e (molecule on the surface) to −0.54e (molecule at step), cf. Supplementary Fig. 4. In contrast, the interaction of the molecule’s dipole with the slightly charged step edges cannot explain the decrease of the binding energy. The complex has a dipole along the axis of the Re–Cl bond of about *P* = −0.276 e Å or −1.32 Da with respect to the Re atom and the metal atoms close to the step edge show only little charging of about 0.01e per atom. Also, the binding of the bipyridine to the surface plays only a minor role, as the rotational orientation of the molecule with respect to the step edge does not affect the binding energy.

We conclude that the strong affinity between the *fac*-Re(bpy)(CO)_3_Cl molecule and the 〈110〉 step edge is the driving force to explain the experimental observation that the formation of *fac*-Re(bpy)(CO)_3_Cl clusters always starts at 〈110〉 steps (Fig. 2a).

On the one hand, the DFT analysis of a single *fac*-Re(bpy)(CO)_3_Cl molecule attached to a step does not provide a preferential configuration for the complex relative to the surface, as evident by the hardly varying binding energies in Fig. 4c–e. But on the other hand, the experimental data clearly indicate that only one ordered cluster structure is found at the steps, for (i) isolated clusters, for (ii) extended structures fully decorating step edges and for (iii) completely covered surface areas.

### The structure of 1D and 2D molecular clusters

To refine the model, the structures of quasi-one-dimensional chains as well as of monolayers were evaluated on basis of experimental data, the detailed discussion is given in Supplementary Note 3. We find that the chain structure is relatively robust and thus not affected by the attachment of a monolayer on either of the adjacent terraces. On the basis of this analysis, we conclude that the configuration in Fig. 4e is the one used to build up the 1D molecular nanowires and thereby also the starting point for the 2D monolayers.

The molecular arrangement within a monolayer on the flat silver surface was analyzed on the basis of high-resolution topographies as shown in Fig. 5c, d. Based on the simulated constant current topography in Fig. 4a we attribute every topographic maximum to be belonging to one molecule and, in a first step, propose a simple model for the underlying molecular structure as shown in Fig. 5e. The obvious periodicity of the layer originates in a column-wise rotation of the complex, as is indicated by the colored arrows in Fig. 5e, repeating itself after four molecules.

Taking into account that the chloride ion is facing the surface (cf. Fig. 4), we propose a possible molecular arrangement on the Ag(001) surface and for a monolayer being attached to an occupied step edge, as shown in Fig. 5a, b. In the monolayer, each unit of four molecules is separated from the next by a gap of a few rows of uncovered silver (between the red and green molecules in Fig. 5e). The interaction of neighboring molecules is likely to be governed by van der Waals forces. The neighboring complexes do not form chemical bonds and the interaction energy E of neighboring dipoles (distance of *r* ~ 0.5 nm) is firstly repulsive and secondly also rather small, $E = 2P^2/\left( {4\pi \varepsilon _0r^3} \right)\sim 0.02\,{{{{{{{\mathrm{eV}}}}}}}}$, with the vacuum permittivity *ε*_0_.

While we are aware that alternative models can be envisioned, the simulated STM images are consistent with the experimental results, supporting the rationality of the proposed atomic configuration for the molecular monolayer.

### Growth model

The key findings of our study is a one-dimensional faceting of decorated step edges which in turn is the cornerstone of the growth of highly oriented monolayers. Combining the experimental with DFT results, we propose the following growth model for *fac*-Re(bpy)(CO)_3_Cl on Ag(001) at 300 K substrate temperature.

### Nucleation of molecular clusters

We would like to start by mentioning that the overall coverage of the surface does not represent the amount of evaporated and deposited molecules. As on many terraces, full monolayer coverage is found, obviously, enough molecules have been deposited to achieve monolayer coverage. The impact of diffusion of the molecules on the surface as a possible origin of the observed inhomogeneity is discussed in detail in Supplementary Note 4. But from the strong variations of the local surface coverage, we conclude that desorption of the molecules from the surface plays an important role in suppressing nucleation of small clusters on the free surface. Instead, the experimental, as well as the DFT results, provide strong evidence for nucleation to start at steps.

In addition, even for low coverage but also for completely covered surfaces we observe a clear preferential step orientation of the occupied segments along 〈110〉 (e.g. Figs. 2a and 3b). Regarding the highly inhomogeneous coverage on the atomic scale as well as the experimental observation that already small isolated clusters are found at the preferential step edge configuration running along the 〈110〉 direction, it is most likely that nucleation happens at such specific sites, i.e. the growth starts with molecules diffusing to steps and attaching at segments having the preferred local orientation along the crystal direction [110]. The minimum observed number of molecules in a cluster is four implying that several molecules are necessary to form a stable nucleus.

### Molecule-step edge interaction

One of the central experimental findings of this study is that the overwhelming majority of the covered step edges show a perfect alignment along the directions 〈110〉. In contrast, on the uncovered silver surface, we do not find any comparable alignment of the step edges, (cf. Supplementary Fig. 2). This alignment is not only found on the local scale, as it is found for steps that are deviating from the directions 〈110〉 (cf. Fig. 2b). It is also found on larger scales, for example, shown in Fig. 2c. For a covered sample that was held at room temperature for several days, we even find ordered molecular structures at quasi perfectly aligned step edges with up to a hundred nanometers length (cf. Supplementary Fig. 3).

As the samples are at room temperature during sublimation, not only the molecules but also Ag atoms are highly mobile. While it was reported that the most efficient mass transport channel causing fluctuations of silver steps on clean surfaces is the exchange of atoms between step and surface^28^, other findings suggest the most efficient mass transport channel on clean metal surfaces to run alongside step edges^29,30^. In any case, it is clear that there are diffusion-driven mechanisms allowing a rearrangement of steps on metal surfaces. Hence, we conclude that there is a strong link between the molecular cluster growth and the reorientation of the steps by silver atom diffusion.

In the literature, adsorbate-induced restructuring of a surface is referred to as faceting^31,32^. The significance of faceting to molecular growth has been shown in studies of different dimensional molecular growth, for example in 2D for thin-film growth of C_60_ on Pd(110)^33^, or the growth of alkane layers on Cu(100)^16,17^. Furthermore, faceting is found for lower dimensional growth, e.g. in 1D for the anchoring of molecular chains to a surface^18^. On the single-molecule scale (0D), e.g., the adsorption of the Lander molecule has been investigated^34–36^. It was found that besides adsorbing only at specific surface sites the molecule promotes the growth of surface structures fitting the molecule’s dimensions. For the present model system, we can show that the interaction between molecules and substrate results in low-dimensional faceting that is an essential prerequisite for the growth of well-ordered molecular structures in 1D, 2D, and 3D. It is fair to say that our data do not provide access to the exact atomistic processes leading to the reorientation of the step edges.

### Monolayer growth

Since we have not observed any pronounced molecular islands on the terraces, nucleation on the free surface is negligible. Instead, small monolayers areas attached to molecules decorating step edges (Fig. 3a, indicated by the blue arrows) suggest that the decorated step edges are the nucleation sites for monolayer growth. Besides, all the monolayer nuclei are found at step regions well-aligned along the crystal’s symmetry directions 〈110〉. We conclude that the nucleation of the molecular monolayer dominantly happens at completely occupied, for tens of nanometers-oriented step edges and not at randomly oriented step edges. Secondly, a monolayer exclusively grows on the upper part of the steps, i.e. no growth is observed on the bottom of a step. Thereby, the formation of grain boundaries resulting from growth starting either at the lower or the upper flanking step edge^37^ is strictly avoided.

These very specific nucleation processes found for both 1D clusters and 2D monolayers explain the inhomogeneous coverage of the surface by molecular clusters. For the pristine surface, appropriate binding sites at steps having 〈110〉 orientation are rather limited and at differing sites molecules will desorb with a higher probability from the surface. This is evident, e.g., by the large difference in binding energies for molecules on the undisturbed surface in contrast to molecules at step edges. And, if the fully decorated step edges are not perfectly aligned along the directions 〈110〉 also the lack of appropriate nucleation sites for the monolayer will increase the likelihood of desorption, which means that defects in the 〈110〉 oriented steps are able to prevent further growth. This is visible, e.g. in Supplementary Fig. 9.

In addition, we find the local step density to be an important growth parameter. In surface regions of high step density, we find an additional form of surface faceting promoted through self-assembled molecular clusters. A topography showing several well-ordered clusters is discussed in Supplementary Note 5. The molecules form a periodic structure along the crystal direction [210]. On a very local scale, the [210] steps consist of short [110] step segments and, hence, offer similar binding sites that fit the molecular dimensions. This, again underlines that the crystal symmetry axis [110] is geometrically preferred by the molecule and shows a complex growth mode that is only dominant in a suitable substrate environment.

## Conclusion

In summary, our results present a comprehensive picture of the deposition and self-assembly of thermally stable rhenium complexes on the Ag(001) surface. All steps in the molecular growth of *fac*-Re(bpy)(CO)_3_Cl on silver rely on the availability of step edges aligned along 〈110〉. Using large-scale DFT calculations the growth hierarchy can be understood and interpreted in terms of the underlying molecular structures and the corresponding binding energies. Rearrangement of the substrate atoms is involved in the molecular cluster growth affecting the local step orientation. This promotes the formation of well-ordered structures. The resulting long-range ordered 1D molecular wires are found to be the prerequisite for 2D growth resulting in long-range ordered molecular monolayers and finally for growing 3D structures. Our results show how well-designed surface morphology can be used to guide and control molecular self-assembly in 1D, 2D as well as 3D.

## Methods

### Sample preparation

The Ag(001) surface was cleaned by repeated Ar^+^-sputtering and annealing cycles resulting in a stepped surface with random step orientations and inhomogeneous terrace widths. Sublimation of the *fac*-Re(bpy)(CO)_3_Cl (bpy = 2,2′-bipyridine) complex (see Fig. 1a) onto the clean silver was done under ultra-high vacuum (UHV) conditions (pressure *p* < 10^–9^ mbar) with the substrate at room temperature.

### Scanning tunneling microscopy

The formation of a molecule film of *fac*-Re(bpy)(CO)_3_Cl (bpy = 2,2′-bipyridine) on the noble metal surface Ag(001) was studied by STM. After the preparation, the crystal was transferred into a home-built cryogenic STM under UHV (*p* < 5 × 10^–11^ mbar). The measurements were performed in constant current mode at temperatures of 80 K as well as of 8 K. Constant current topographies are referred to as topographies in the main text. For better visibility, in the topographic data physically flat areas were fitted to a plane. If useful, data were interpolated, line averages were subtracted, and 3 × 1 median filters were applied.

### Synthesis of the complex

For the synthesis of the complex, we followed the procedure as described previously^38^. To a solution of 2,2´-bipyridine (86.4 mg, 553 μmol, 1.01 eq.) in dry toluene (10 mL) pentacarbonylchloridorhenium (199 mg, 549 μmol, 1.00 eq.) was added and the mixture heated to 100 °C for 6 h. After cooling down to room temperature the solvent was removed under reduced pressure. The yellow residue was washed with diethyl ether (3 × 5 mL) and hexane (3 × 5 mL). Drying in vacuum yielded the desired product (233 mg, 504 μmol, 92%) as a yellow solid. Prior to STM studies the *fac*-Re(bpy)(CO)_3_Cl was further purified by sublimation at about 200 °C in HV (*p* < 10^–3^ mbar). Once in the preparation chamber, the molecular source was degassed at 150 °C for 3 days under HV (*p* < 10^–6^ mbar).

### Sum-frequency generation (SFG)

Experimental details to measure vibrational SFG can be found in Supplementary Note 6. In short, tunable 20 ps IR pump and probe pulses, as well as 532 nm laser pulses, were used in order to study the generated SFG signal for pump pulses in the range of the molecule’s characteristic CO absorption bands at roughly 2000 cm^−1^ on a monolayer of the rhenium complex on the Ag(001) surface. The SFG response (*I*_pump_) is measured at a fixed time delay Δ*t* after the IR_pump_ pulse has excited the system at a certain wavenumber and normalized to the signal without a previous pump pulse (*I*_0_). The pulse scheme is depicted as an inset of Fig. 1c. The *fac*-Re(bpy)(CO)_3_Cl monolayer covered Ag(001) samples were prepared under UHV conditions, the SFG measurements were carried out under ambient conditions.

### Density functional theory

The calculations were performed to explore the geometric configurations of *fac*-Re(bpy)(CO)_3_Cl molecules on the Ag(001) surface with and without step edges, the binding energies of a *fac*-Re(bpy)(CO)_3_Cl molecule at different sites, and various patterns of *fac*-Re(bpy)(CO)_3_Cl molecules on Ag(001) surfaces. All the DFT calculations were carried out with the Vienna ab initio Simulation Package (VASP)^39^ based on DFT with the van der Waals (vdW) interactions in terms of semi-empirical DFT-D2 correction^40^. The used exchange-correlation function is the generalized gradient approximation (GGA) of Perdew–Burke–Ernzerhof (PBE)^41^ and the electronic plane wave interception energy was set to be 400 eV. To avoid interactions between the neighboring periodic images, a vacuum layer larger than 15 Å was used in modeling. In structure optimizations, the energy convergence criteria of 10^–5^ eV and force convergence criteria of 0.01 eV per Å are used. The binding energies (*E*_b_) of a *fac*-Re(bpy)(CO)_3_Cl molecule on Ag(001) surface were calculated as:

$$E_{{{{{{{\mathrm{b}}}}}}}} = \left( {E_{{{{{{{{\mathrm{system}}}}}}}}} - E_{{{{{{{{\mathrm{substrate}}}}}}}}} - n \cdot E_{{{{{{{{\mathrm{molecule}}}}}}}}}} \right)/n$$

where *E*_system_ and *E*_substrate_ are the calculated energies of the whole system and the Ag substrate, respectively. *E*_molecule_ is the energy of one molecule and n denotes the number of molecules in one unit cell of the computational model.

As discussed elsewhere^42,43^, the simulated STM images based on DFT calculations do not correspond to a current value as that in real experiments. Instead, we calculate the 3D charge density distribution and the simulated constant current image corresponds to an iso-value surface.

All the STM images for multiple molecules grown on Ag surfaces are constant height images and were obtained at heights of 6 Å above the metal surface (maximum of *z*-coordinate).

## Supplementary information

Supplementary Information

Description of Additional Supplementary Files

Supplementary Data 1

## Data Availability

The data that support the findings of this study are available from the corresponding author upon reasonable request. Besides, the optimized structure of the single complex in a vacuum is provided in Supplementary Data 1.
