# Supplementary material for: Ordering a rhenium catalyst on Ag(001) through molecule-surface step interaction
Source: Commun Chem. 2022 Jan 10;5:3. doi: 10.1038/s42004-021-00617-9 (PMC9814538; doi:10.1038/s42004-021-00617-9)
Supplement: Supplementary file 1 — Supplementary Information [file 42004_2021_617_MOESM1_ESM.pdf]

Supplementary Information

Ordering a rhenium catalyst on Ag(001) through molecule-surface step interaction

Ole Bunjes[1], Lucas A. Paul[2], Xinyue Dai[3], Hongyan Jiang[4], Tobias Claus[1], Alexandra Rittmeier[1], Dirk Schwarzer[4], Feng Ding[3,5], Inke Siewert[2] and Martin Wenderoth[1*]

[1] IV. Physikalisches Institut, Georg-August-Universität Göttingen, Friedrich-Hund-Platz 1, 37077 Göttingen, Germany.

[2] Institut für Anorganische Chemie, Georg-August-Universität Göttingen, Tammannstraße 4, 37077 Göttingen, Germany.

[3] Center for Multidimensional Carbon Materials, Institute for Basic Science (IBS), Ulsan 44919, Republic of Korea.

[4] Department of Dynamics at Surfaces, Max-Planck Institute for Biophysical Chemistry, Am Faßberg 11, 37077 Göttingen, Germany.

[5] Department of Materials Science and Engineering, Ulsan National Institute of Science and Technology (UNIST), Ulsan 44919, Republic of Korea

*email: martin.wenderoth@uni-goettingen.de

Supplementary Note 1: Preparation of silver wool for test evaporation

Before the test evaporation onto silver wool the wool was washed with acetone and water. Subsequently the silver wool was purified electrochemically by first applying 0 V vs. a saturated calomel electrode (SCE) in aqueous KCl (0.1 M) solution for five minutes, then -0.3 V vs. SCE for five minutes and -0,4 V vs. SCE for 10 minutes. Afterwards, the silver was rinsed with millipore water, dried in vacuum and used immediately after purification. Finally, the silver wool was cleaned by sputtering with Ar^{+} ions in-situ. Then the complex was deposited using very similar conditions as during the sublimation onto the Ag-crystal for the STM measurements. After deposition, the molecules were ex-situ washed out of the wool using acetone and dried.

Supplementary Note 2: Static SFG measurements

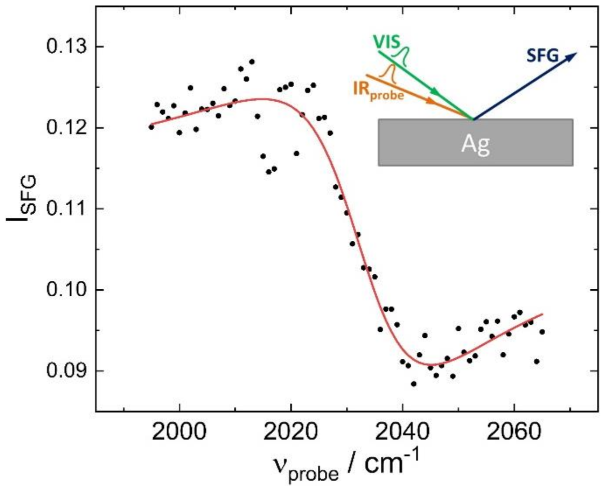

Supplementary Figure 1: SFG spectrum (IR_{pump} blocked) of the A’(1) CO stretching mode of *fac*-Re(bpy)$(CO)_3$Cl on Ag(001). The fit gives a centre frequency of 2033 ± 6 cm^{-1}.

The measured SFG spectrum shown in Supplementary Fig. 1 was fitted with the following function

$$I_{SFG} \propto \left|\chi_{NR}^{(2)} + \chi_{R}^{(2)}\right|^2 = \left|\left|\chi_{NR}^{(2)}\right| + \frac{a_j}{(\omega_j - \omega - i\Gamma_j)} e^{-i\varphi_j}\right|^2,$$

where $\chi_{NR}^{(2)}$ and $\chi_{R}^{(2)}$ are the non-resonant (from the Ag surface) and resonant (from the carbonyl stretching mode) contribution to the surface’s second-order susceptibility, respectively. The resonant part is defined with a_j, ω_j, and Γ_j as oscillator strength, resonance frequency, and damping constant of the vibrational mode j, respectively. φ_j is the phase difference between the non-resonant and resonant contributions.

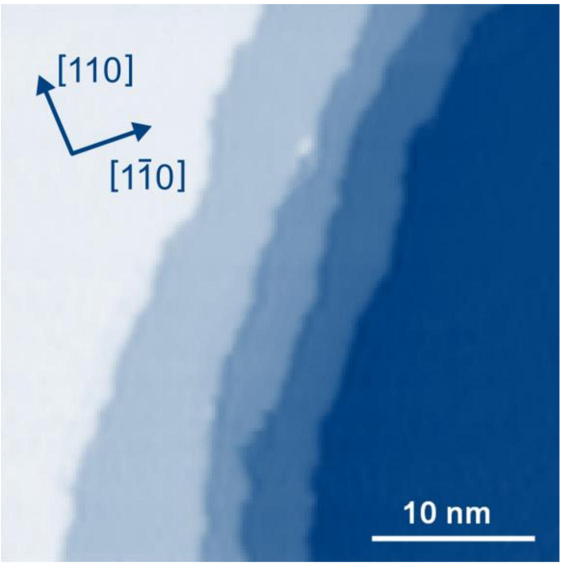

Supplementary Figure 2: Constant current topography (I_{set} = 50 pA, U_{bias} = 2 V and T = 8 K) of the pristine Ag(001) surface showing a few neighboring silver terraces. As in Fig. 2(b), the average crystal direction of the step edges differs from the high symmetry directions [1$\bar{1}$0] and [110], both directions are indicated in the image.

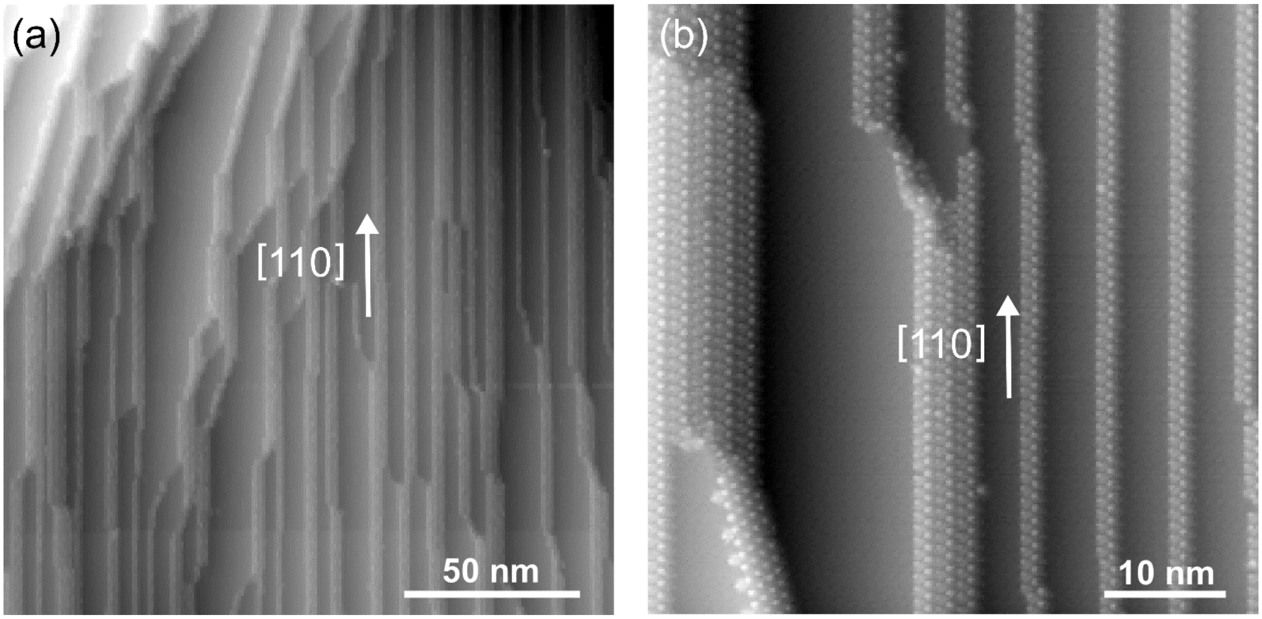

Supplementary Figure 3: Long-range ordered molecular wires at step edges. (a) Topography (U_{bias} = 2 V) showing silver step edges that are almost perfectly aligned along the silver crystal direction [110] for a few hundred nanometers. After deposition of the molecules the sample has been at room temperature for about a week. (b) High resolution image (U_{bias} = 1 V) of almost perfectly grown molecular wires. The tunneling parameters for both datasets are I_{set} = 50 pA, and T = 80 K. Data are colored such that all the molecular clusters appear bright and are easily distinguishable from the silver substrate.

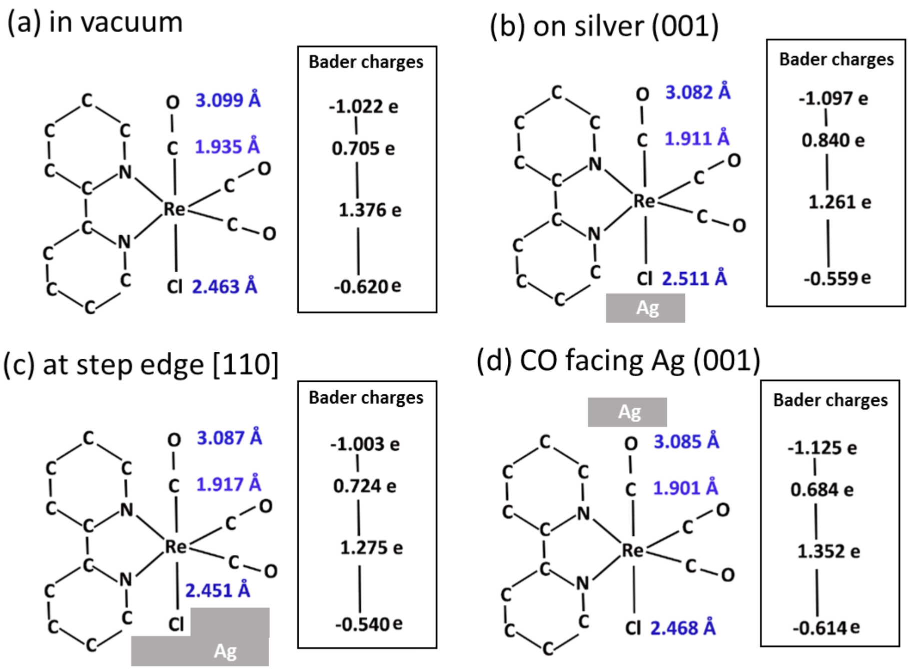

Supplementary Figure 4: Bader charge distributions of a few key atoms along the z-direction of the complex. (a) The complex in vacuum. (b) The complex on the surface with chloride ion facing the substrate. (c) The complex with the Cl ligand attached to a monatomic [110] step edge. (d) The complex on the surface with CO facing the substrate. The blue texts indicate the distances of O, C and Cl to the Re. The corresponding Bader charges of the same atoms are given in the box on the right of each configuration.

Supplementary Note 3: Experimental findings on the structure of molecular nanowires and of attaching monolayers

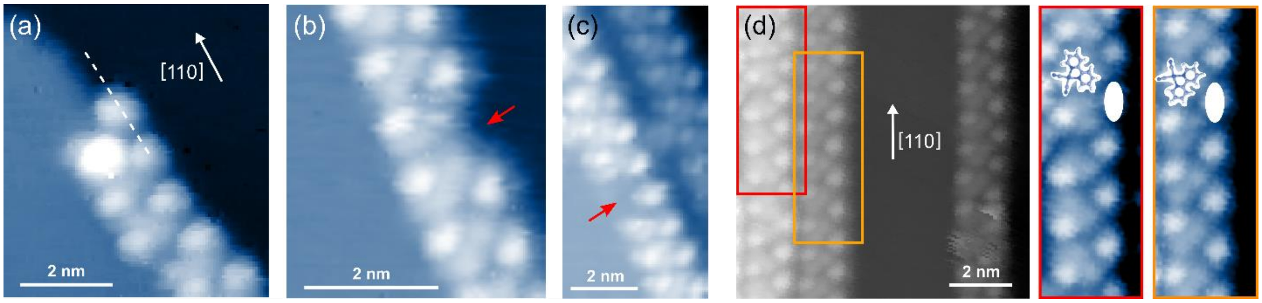

Supplementary Figure 5: Structure of one-dimensional molecular chains decorating step edges. (a) End of molecular chain. The chain consists of two maxima that are found alternatingly one more on the upper the other one more on the lower flanking terrace, the latter is indicated by the white dashed line. (b) Defect in chain showing a missing maximum on the lower terrace, indicated by the red arrow. (c) Defect in a chain showing a missing maximum on the upper terrace, indicated by the red arrow. (d) Surface area in which all the visible step edges are decorated by molecular chains. Framed in red and in orange are magnifications of two neighboring chains that show rotational symmetry of the upper structure by about 90° with respect to the surface normal. With the help of the simulated constant current image in Fig. 4(a) this can be identified to be one molecule as indicated by the structural model of the complex. The maximum on the lower terrace seems to have a symmetry axis parallel to the step edge direction (white oval). The tunneling parameters for all datasets are I_{set} = 50 pA, U_{bias} = 2 V and T = 80 K.

In Supplementary Fig. 5(a) one can see the end of a molecular chain decorating a step edge. The chain consists of two kinds of topographic maxima that alternatingly decorate the step edge. While the maxima on the left side are more located on the upper flanking terrace, the maxima on the right side are more positioned on the lower flanking terrace, as indicated by the white dashed line. We identify the smallest building blocks of the chain by analyzing the defects shown in (b) and (c). In (b) one can see a missing maximum located more on the upper flanking terrace, while in (c) one can see a missing maximum that is more located on the lower flanking terrace. This suggests that each of the maxima that are building up the molecular chains is a single molecule. We analyze the symmetry of both molecules separately with the help of Supplementary Fig. 5(d). In the figure a surface area is shown in which all visible silver steps are completely occupied by molecular chains. Magnifications of two of the shown chains can be seen with comparable color scales framed in red and orange on the right. While the molecules more located on the lower terrace (indicated by the white oval) show a symmetry along the step edge, the molecules on the upper terraces show a symmetry that is rotated by 45° with respect to the step edge. Framed in red and orange the two found options in which the upper molecules are rotated by roughly 90° with respect to each other are shown, indicating that both possible arrangements are energetically equally favorable. Taking into account the simulated constant current topography of an isolated molecule in Fig. 4(a) we identify the upper molecule to be lying on the upper flanking terrace as depicted by the structural model of the complex. The topographic maximum is found to be located on the CO group that is facing up from the complex. For the second molecule, that seems to be more positioned on the lower flanking terrace one may identify two possible geometrical orientations. These are shown in Supplementary Fig. 6.

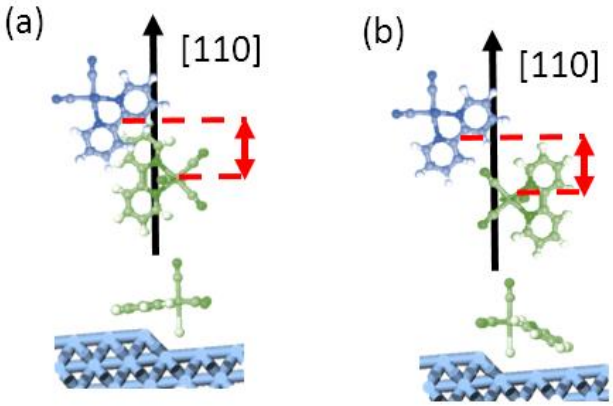

Supplementary Figure 6: Possible geometric orientation of molecule (green) on the lower flanking terrace of a covered step edge in a ball-and-stick model. (a) Bipyridine is located on the upper terrace while the chloride ion is located on the lower terrace. (b) Both chloride and bipyridine are located on the lower terrace.

The molecule shown in green is the one of which the orientation is going to be discussed. Obeying the symmetry along the step edge found in Supplementary Fig. 5(d), taking into account the simulation result that it is energetically more favorable to have the chloride ion facing the surface as given and keeping in mind that the molecule in question is found to be more located on the lower flaking terrace (cf. Supplementary Fig. 5(a)), there are two possible arrangements of the green molecule. The question is which parts of the molecule are located on the upper flanking terrace and which are on the lower? The configuration in which only the bipyridine is on the upper terrace is shown in (a). Part (b) shows the molecule being completely on the lower terrace. These correspond to the simulations of the single molecule at the step edge from Fig. 4(c) and (e), respectively. We exclude the configurations shown in Fig. 4(d) as this does not fit the observed symmetry as well as the one shown in Fig. 4(f) as this is energetically not favorable. The monolayer structure can be simulated without the requirement of close proximity between the bipyridine parts of two neighboring complexes (cf. Fig. 5(a)) which would be necessary for configuration shown in Supplementary Fig. 6(a), i.e. for the 1D chain structure we favor the option shown in Supplementary Fig. 6(b).

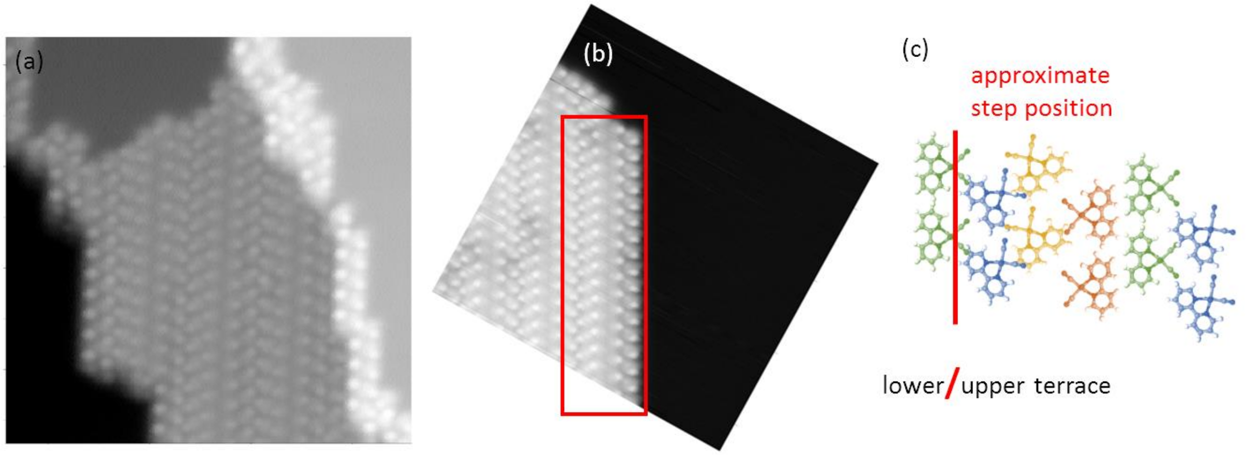

Supplementary Figure 7: Structure of monolayer attached to step edge. (a) Topography (I_{set} = 50 pA, U_{bias} = 0.2 V and T = 80 K) of surface area showing a monolayer that is attached to a step edge. (b) Border between molecular monolayer and unoccupied silver surface, (I_{set} = 50 pA, U_{bias} = 2 V and T = 80 K). The molecules of the determined surface unit cell that lie on the upper terrace are framed in red. (c) Ball-and-stick model of molecular monolayer attached to a silver step in top view.

The structures of the molecular chains is not affected by a monolayer that attaches to them on either of the two flanking terraces. Nonetheless the chain structure is strongly linked to the structure within the monolayer. This can be exemplarily seen in Supplementary Fig. 7(a) as the ordered sections of the shown molecular chains show the same structures though one has a monolayer attaching on the upper flanking terrace and the other one has the monolayer attaching on the lower flanking terrace. We conclude the chains to be comparably robust. The structure of the monolayer growing from the lower step is a continuation of the chain structure except the molecule that is located on the lower terrace (shown in green in Supplementary Fig. 6). For the transition of the monolayer to the unoccupied terrace we dominantly find the situation that can be seen in Supplementary Fig. 7(b), allowing us to construct the unit cell as shown in (c). Besides, this suggests that the structure built by the green and the blue molecules is robust not only in the 1D chains but also in the 2D layers.

Supplementary Note 4: Contribution of diffusion to the inhomogeneous surface coverage

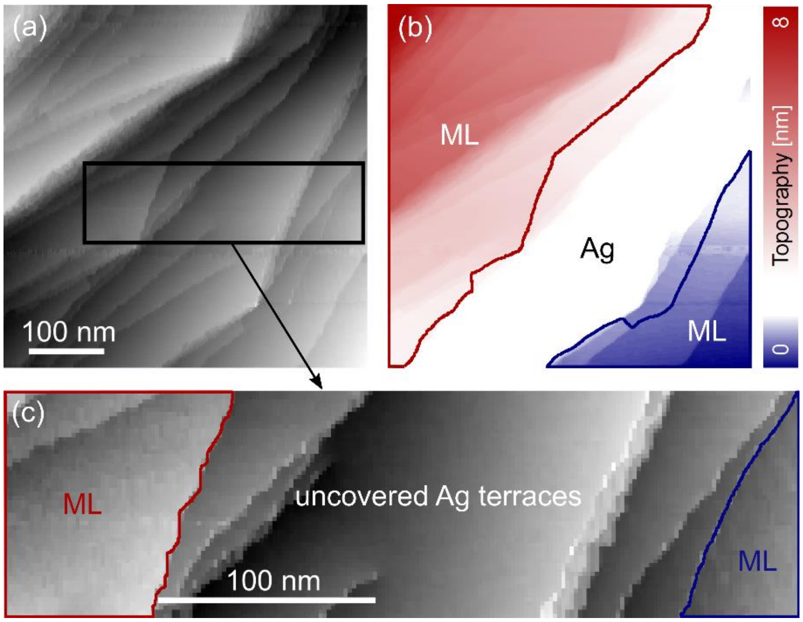

Supplementary Figure 8: Inhomogeneous surface coverage. (a) Topography (I_{set} = 50 pA, U_{bias} = 2 V and T = 80 K). (a) Large scale topography showing several terraces. Data are colored such that all the step edges are visible. (b) The same topography as in (a). The highest terraces (framed in red) as well as the lowest terraces (framed in blue) are largely covered by molecular monolayers (ML). In contrast the center region remains uncovered by molecules except for the step edges. (c) Magnification of region framed in black in (a) showing the width of the unoccupied region between the regions framed in red and in blue. Data are colored such that all the step edges are visible. As a side note, the unoccupied silver surface shows a different noise characteristic compared to the surface area that is occupied by molecules, e.g. framed in red.

In the main manuscript we have concluded that monolayer growth of *fac*-Re(bpy)$(CO)_3$Cl on Ag(001) requires surface steps running in (110) direction and that desorption might play a dominant role. In this part we would like to extend the discussion on how diffusion of *fac*-Re(bpy)$(CO)_3$Cl molecules can be included in the growth model. As we do not observe diffusion in a direct manner, our discussion on diffusion is based on firstly the strongly inhomogeneous growth of monolayers on neighboring terraces (cf. Fig. 3(a) and Supplementary Fig. 8) in combination with secondly the observation of clusters aggregating at steps, (cf. Fig. 2(a)).

While in the central manuscript we propose that desorption has a great impact, for the following discussion, let us assume that desorption of molecules on the molecular growth is negligible and the strong inhomogeneous coverage is the result of diffusion of *fac*-Re(bpy)$(CO)_3$Cl on large distances.

The discussion has to distinguish between transport across steps and no transport across steps.

Case one: Diffusion across step edges can be neglected

In consequence, all molecules adsorbing on a given terrace have to stay on this terrace. Neglecting desorption, one has to discuss the diffusion of the molecules within a certain terrace as the dominant mechanism for the observed growth behavior. Without a dominant contribution from inter terrace diffusion, the number of molecules (or the area occupied by the molecular clusters) on a terrace should be proportional to the area of the silver terrace in question. This disagrees with the experimental observations, as can be seen, e.g., in Fig. 3(a). The center terrace is largely occupied while the neighboring terraces hardly show any occupation by molecular monolayers. In more detail, from overview topographies we know that the largely occupied terrace and the terrace directly on its left have similar areas, but both terraces show clear differences concerning their occupation with molecules.

In conclusion, the assumption that both desorption from the surface as well as diffusion across steps do not play a role contradicts the experimental observations.

Case two: Diffusion across steps edges dominantly contributes.

Experimentally, the inhomogeneous coverage is observed on a length scale on which the average number of deposited molecules can be assumed to be constant. During the measurements one regularly finds surface areas on which all terraces are almost completely covered by molecular monolayers on a scale of 500*500 nm². These areas are coexisting with surface areas that are very non-homogeneously covered. One example for the latter is shown in Supplementary Fig. 8. In Supplementary Fig. 8(b), the regions that are colored and framed in red and blue are largely occupied by molecular monolayers, whereas the central terraces in between both areas are not covered. This region has a width of more than 200 nm, as can be seen in Supplementary Fig. 8(c). This implies, if the molecules were not desorbing, but diffusing away from the center and attaching to a molecular cluster on a different terrace (inter terrace diffusion) these molecules would need to diffuse at least 100 nm on the surface. If they were to diffuse out of the visible surface area within the same terrace they would need to diffuse several hundreds of nanometers on the surface. This in combination with the preferred nucleation site at the [110] steps, underlined for example by the monolayer nuclei seen in Supplementary Fig. 9, leads us to the assumption that desorption considerably influences the growth of molecular clusters, such that we discuss its impact as the dominating factor.

Nevertheless, we do not completely exclude the possibility of diffusion to contribute. If one assumes diffusion across step edges with a very long diffusion length this might be a possible origin for the inhomogeneous surface coverage.

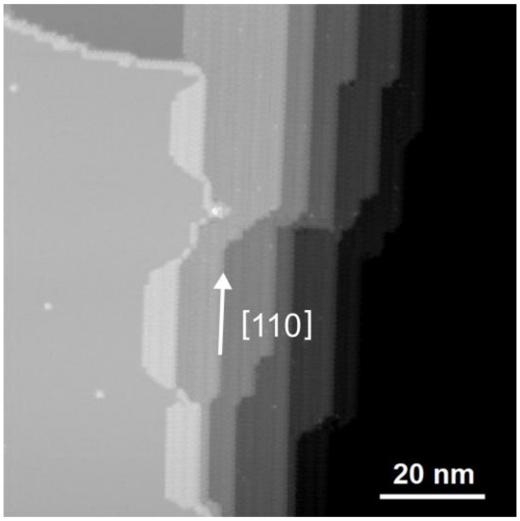

Supplementary Figure 9: Monolayer growth at oriented step edges is stopped at defects. The monolayer patches on the topmost silver terrace (left) stop at defects in the occupied step edge. Tunneling parameters are I_{set} = 50 pA, U_{bias} = 2 V and T = 80 K.

Supplementary Note 5: Molecular clusters in areas with high step density

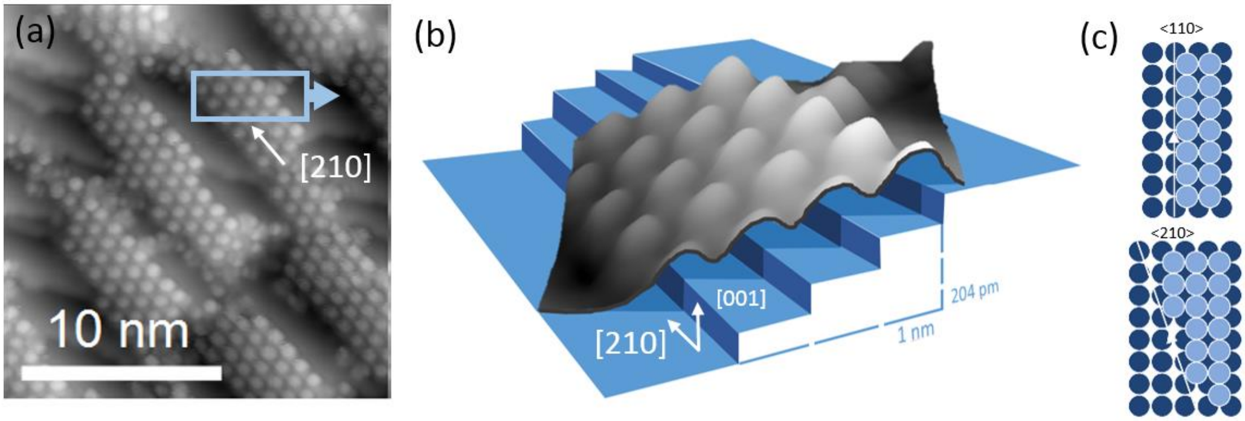

Supplementary Figure 10: Molecule induced alignment of Ag steps is also found along the [210] crystal direction in step-rich surface regions. (a) Constant current topography (I_{set} = 50 pA, U_{bias} = 2 V and T = 8 K) of several molecular fields. Data are colored such that all the molecular clusters appear bright and are easily distinguishable from the silver substrate (dark grey). Each row of topographic maxima (white) corresponds to a single silver step edge parallel to the white arrow marking the crystal direction [210]. The contour shown in (b) is marked by the light blue rectangle. (b) 3D representation of a molecular field section (b/w) including a schematic model of the underlying silver steps (blue). The molecular cluster is covering several equally spaced silver step edges running along the crystal direction [210]. Each step has a width of about one nanometer and a height of 204 pm. (c) The ball models depict the top view onto the atomic structure of step edges on the Ag(001) surface running along the crystal directions [110] and [210].

In surface regions of high step density, we find a special case of surface reordering promoted through self-assembled molecular clusters. A topography showing several well-ordered clusters is shown in Supplementary Fig. 10(a). The molecules form a periodic structure along the crystal direction [210]. As the topographic maxima are indistinguishable from each other within the precision of our measurement, we conclude that the molecule-surface interaction, and hence the underlying surface configuration is equal for all identical parts of the molecular structure. This leads to the conclusion that beneath the molecules there are three equally spaced silver terraces each having a width of about a nanometer, cf. Supplementary Fig. 10(b). Supplementary Fig. 10(c) shows a ball model of steps running along the directions [110] and [210]. It is visible that the latter consists of short subsequent segments of [110] step edges. Along the [210] direction, geometrically equal binding sites are separated by 9.1 Å, which is about the maximum length to be found in the molecular geometry (cf. Fig. 1(a)).

Supplementary Note 6: Vibrational sum-frequency generation (SFG) measurements

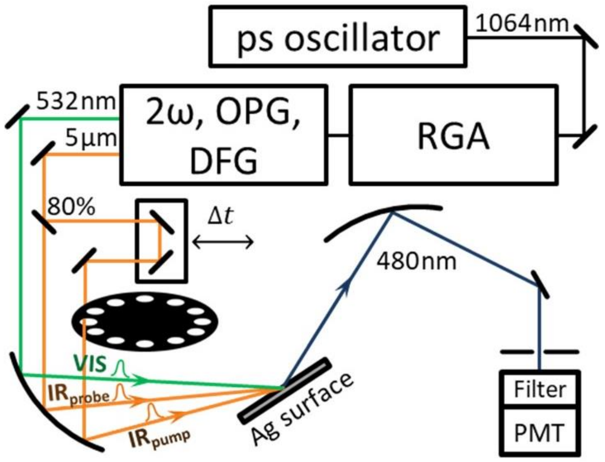

Supplementary Figure 11: Experimental setup to measure SFG spectra (IR_{pump} blocked) and IR_{pump} pulse induced changes of the SFG response of *fac*-Re(bpy)$(CO)_3$Cl on Ag(001).

Experimental details to measure vibrational sum-frequency generation (SFG) spectra have been reported before [1], the setup is shown schematically in Supplementary Fig. 11. Tunable IR and 532 nm upconversion pulses were generated with a 50 Hz Nd:YAG based oscillator/regenerative system with subsequent optical parametric and difference frequency generator (PL2231-PG511-DFG, EKSPLA) providing 20 ps pulses at around 2000 cm^{-1} with a bandwidth of 2 cm^{-1}. To record conventional SFG spectra 5 μJ IR (IR_{probe}) and 10 μJ 532 nm pulses were focused onto the sample, overlapped in time and space, and the generated SFG light intensity at ~480 nm was recorded while tuning the IR_{probe} frequency. Additionally, changes in the SFG response induced by an IR pump pulse (IR_{pump}, with 30 μJ pulse energy from the same optical parametric generator as IR_{probe}) exciting the sample 15 ps prior to the IR_{probe}/532 nm pulse pair were measured. When IR_{pump} is resonant with a vibrational transition the SFG response changes due to population transfer from the vibrational ground to the excited state. The pump-induced change in the SFG spectrum was measured by blocking every second pump pulse with a synchronized chopper, recording the intensity with (I_{pump}) and without IR_{pump} (I_0) excitation, and plotting the intensity ratio I_{pump} I_0^{-1} vs. IR frequency. These IR_{pump} induced SFG spectra turn out to be less noisy and produce more narrow spectral features than conventional SFG spectra (see Ref. [1]). All three laser pulses were p-polarized with respect to the plane of incidence.

Supplementary Reference

[1] S. Kumar, H. Jiang, M. Schwarzer, A. Kandratsenka, D. Schwarzer, and A. M. Wodtke, Phys. Rev. Lett. **123**, 156101 (2019).
