# Supplementary material for: Ordering a rhenium catalyst on Ag(001) through molecule-surface step interaction
Source: Commun Chem. 2022 Jan 10;5:3. doi: 10.1038/s42004-021-00617-9 (PMC9814538; doi:10.1038/s42004-021-00617-9)
Supplement: Supplementary file 2 — Description of Additional Supplementary Files [file 42004_2021_617_MOESM2_ESM.pdf]

Description of Additional Supplementary Files

File Name: Supplementary Data 1

Description: Structure of optimized single molecule in vacuum
